# Book Reviews

**Published:** 1802-08-01

**Authors:** 


### CRITICAL ANALYSIS

OF THE

### RECENT PUBLICATIONS

ON THE DIFFERENT BRANCHES OF

### PHYSIC, SURGERY, & MEDICAL PHILOSOPHY.

---

*Commentaries on the History and Cure of Diseases;* by WILLIAM HEBERDEN, M. D. 8vo. 483 pages. 1802.

THE Medical Public will doubtlefs feel confiderable intereft in perufing thefe remains of a late venerable and excellent character, who had fo juftly attained that high eminence in his profeffion, which was the fair reward of extenfive learning, acute obfervation, and an experience acquired by many years of long and active fervices in medical practice.——The author obferves, in his fhort Introduction (and the internal evidence of the whole work confirms his affertion) that "the notes from which the following obfervations were collected, were taken in the chambers of the fick themfelves, or from their attendants. Thefe notes were read over every month; and fuch facts as tended to throw any light upon the hiftory of a diftemper, or the effects of a remedy, were entered in another book, from which were extracted all the particulars here given." This work, therefore, is moftly compofed of *clinical* obfervations, or fuch as occurred to the author when in the actual practice of his profeffion; and they are arranged under the heads of the different difeafes, forming a number of fhort effays, or, as it were, *conversations* upon the feveral fubjects, unconnected with each other, and almoft entirely diverted of theory, or difquifition on the opinions of others. Befides its intrinfic value, the work before us is interefting, as it fhews, in fome degree, the character of the prefent practice of phyfic in this metropolis; the moft ftriking features of which are, cautious judgement, united however with confiderable vigour in the ufe of powerful medicines; a comparative neglect of the purely theoretical part of medicine, arifing not from ignorance in medical learning, but from a wholefome fcepticifm; a rational empiricifm, which gives full fcope to every fair trial of new remedies, and abandons them with equal readinefs when they are unable to ftand their ground by their own merits, or are fupplanted by fome more fortunate rival; a liberal indulgence to the taftes and inclinations of the patient, which, if it in fome inftances leffens the *authority* of the phyfician, increafes his influence; in fhort, a practice directed chiefly to the relief of fymptoms, and profeffional fkill derived from actual obfervation much more than from books.

Our limits will not allow us to examine every ſubject of theſe Commentaries; and, indeed, as diſpaſſionate judgement is the prevailing character of the work, we meet with ſeveral ſections, in which the obſervations which they contain are valuable rather by adding a reſpectable teſtimony to the efficacy of the general mode of practice, than by introducing any new improvement. The firſt ſection is on the ſubject of *diet*, a ſubject which has at all times been much more attended to in other countries than in this, and on which very little ſtreſs is laid by preſent practitioners. Some of our Author's obſervations we ſhall give to our readers. "Many phyſicians appear to be too ſtrict and particular in the rules of diet and regimen which they deliver as proper to be obſerved by all who are ſolicitous either to preſerve or recover their health. The common experience will ſufficiently acquaint one with the ſorts of food which are wholeſome to the generality of men. Whether meat ſhould be boiled or roaſted, or dreſſed in any other plain way, and what ſort of vegetables ſhould be eaten with it, I never yet met with any perſon of common ſenſe (except in an acute illneſs) whom I did not think much fitter to chooſe for himſelf than I was to determine for him." The author then gives a few (and a very few) rules for diet in fever. For drink in this diſeaſe he allows water, either warm or cold, at the option of the ſick perſon, and only in ſuch quantity as is agreeable to him.

The *ratio medendi* is treated of in the ſecond chapter, and we cannot forbear giving briefly the indications laid down by the author, as we believe they are *(in fact)* ſuch as generally direct the operations of the phyſician. The firſt conſideration in the cure of diſeaſe is, whether it requires any evacuation, and what? 2dly. Whether it be a diſtemper for which any ſpecific has been found out? Here the author adds ſome remarks on the uncertainty attending this opinion, but thinks that the title of a ſpecific may juſtly be retained by bark for the cure of agues, quick-ſilver for venereal diſorders, ſulphur for the itch, perhaps opium for ſome ſpaſms, and Bath waters for the injury done to the ſtomach by drinking. 3dly. Vomiting, purging, pain, and other troubleſome ſymptoms, are often ſo urgent as to require immediate relief, for which opium is commonly the moſt effectual means. 4thly. In long and obſtinate diſeaſes, which have reſiſted particular remedies, recourſe muſt be had to the means of generally affecting the ſyſtem, as by mercury, antimony, hemlock, and electrification. Laſtly, where there is room for nothing elſe, the phyſician muſt ſupport the power of life by ſtrengthening the appetite, providing for proper evacuations, &c.——The remarks on *angina* and *scarlet* fever are valuable for the accuracy of obſervation, and judicious directions. The author is decidedly of opinion that the ſcarlet fever and the malignant ſore throat are one and the ſame diſorder, and require exactly the ſame method of treatment; regard being had to the local affection of the throat, ſo much more ſevere in the latter than in the former diſtemper. In the mode of treatment, the author ſpeaks doubtfully of blood-letting, but highly in favour of bliſters, which, he ſays, the patient ſhould never never be without, until he be out of danger. We were rather furprized to find no notice taken of emetics, which are certainly much employed, and are recommended in the higheft terms by an excellent writer on this difeafe, the late Dr. Withering.——The chapter on *gout* is pregnant with good fenfe and found obfervation, and the author very fuccefsfully combats fome of the popular notions concerning this dreadful malady, which have rendered it "the favourite difeafe of the prefent age in England, wifhed for by thofe who have it not, and boafted of by thofe who fancy they have it, though very fincerely lamented by moft who in reality fuffer its tyranny." The author likewife takes occafion to introduce fome of thofe *medico-moral* obfervations which fo naturally fpring from the confideration of certain difeafes, and which flow with peculiar grace from the pen of a venerable and moft refpectable writer, himfelf diftinguifhed for temperance, whofe long and extenfive experience entitle him to the higheft attention.——The author never faw a cafe of *hydrophobia* from the bite of a mad animal, a ftriking proof of the comparatively rare occurrence of this difeafe.——Confiderable attention appears to have been beftowed on the article *ileus, or inflammation of the bowels*, one of the moft formidable of the catalogue of common difeafes, and one in which very much may often be effected by the power of medicine. "The peculiar and diftinguifhing fymptom which characterifes the inflammatory colic in the very beginning," the author obferves, "is a coftivenefs, which it is always extremely difficult and too often impoffible to conquer. As foon as a difcharge downwards can be procured in a copious manner, the patient perceives a quick abatement of all his mifery, and is foon reftored to health." He goes on to obferve, that the evacuation muft be complete, and points out the caufes of fallacy in trufting to an imperfect evacuation which fometimes fupervenes. For the treatment, befides avoiding heating things, applying blifters, fomentations, and above all things ufing purgative medicines, the author ftrongly recommends clyfters of tobacco fmoke, to controul the irregular action of the bowels, and force them to empty their contents in a natural manner. The ufe of opium in this malady has been much controverted, and the author ftates the advantages and objections in a very candid manner. He is ftrongly in favour of their ufe. "Under the protection of an opiate," he obferves, "I have fuccefsfully given more and ftronger purges than would have ftayed without its help; the patient's ftrength has been kept up by fome refrefhing fleeps; and even in hopelefs cafes, in which the dying perfon is harraffed by unfpeakable inquietude, he may be lulled into fome compofure; and without dying at all fooner, he may be enabled to die more eafily."

A caution of high importance is given under the article *Hypochondriacus affectus*. "Many," he obferves, "in a lownefs of fpirits, are not indifpofed to raife them by wine and fpirituous liquors, and they are encouraged and preffed to do it by their well-meaning but ill-judging friends. No words can be too ftrong to paint the danger of fuch a practice in its proper colours. The momentary relief is much too dearly bought by the far greater languor which fucceeds; and the neceffity of increafing the quantity of thefe liquors, in order to obtain the fame effect, irrecoverably ruins the health, and in the moft miferable manner." The author much prefers opium as a cordial. "My own experience," he adds, "has often taught me how fafely and confiftently with bufinefs a courfe of opium may be continued for a confiderable part of man's life, and how practicable it is to be weaned from the habit of it."

We fhall only add, to our review of this valuable work, that we apprehend moft of thofe who perufe it will regret that feveral fubjects of importance are difmiffed with a few curfory obfervations, where fuller particulars of the practice of the late author would have been peculiarly acceptable. We cannot, however, make this any reafonable ground of complaint, as nothing like a fyftematical defcription of difeafes is at all intended to be given, and the Writer excufes himfelf from not having done more in a life of fifty years experience, partly for reafons which will only be attributed to his modefty, and partly from the very flow progrefs which is ever made in the fcience of medicine, and the almoft infuperable obftacles which oppofe our fuccesful refearch into the nature of organized life; with which defponding fentiment the Author takes his leave of the public.

---

*Facts, and some Arguments, tending to shew that the public Decision may with prudence be suspended respecting Inoculation of the Cow-Pox; by* Thomas Lee, a Member of the Univerfity of Edinburgh, 8vo. pp. 36.

The title of the pamphlet before us fufficiently expreffes its contents, namely, to throw a doubt on the inferences drawn from the numerous teftimonies concerning the Cow-pox, which have lately been made the fubject of difcuffion by the medical world in almoft every part of the globe, and in our own country by a felect body of the Legiflature. After fome remarks on the importance of the queftion concerning the efficacy of the Cow-pox, the author makes the following obfervations: "The decifion of a parliament fhould be revered by the people; but parliament, like a jury, can only decide upon evidence; and if the evidence be all one way, of one tenor, can a parliament be blamed, or a jury arraigned, for giving a judgment contrary to the real fact?" He then adverts to what is doubtlefs a fact, that "parliament largely rewarded an old woman for the fecret of difguifing foap in veal broth, as a never failing folvent of ftone in the bladder, and yet the ftone ftill torments mankind;" and that, "the direful tape worm ftill infects us, although Mrs. Boufflers was penfioned and ennobled for communicating a nonfenfical noftrum, which fhe pretended would effectually deftroy it." As we differ confiderably in opinion from the Author before us, we beg to obferve in the firft place, that the late examination before a Committee of the Houfe of Commons on Dr. Jenner's ner's petition, was conducted neither haftily nor partially; that every pains was taken to procure as great a body and variety of evidence as the cafe could admit of; and that if the teftimony there given made fuch a vaft preponderance in favour of Dr. Jenner's difcovery, it furely is to be attributed to fomething more than private friendfhip or the fafhion of the day. The cafes of Mrs. Stephens's medicine and Mrs. Bouffler's fern powder are not exactly fimilar, becaufe being profeffedly fecrets in the hands of the claimants, their merits could not be fo open to inveftigation of every kind, as in the cafe of the Cow-pox, where vaccine inoculation had been already tried with fuccefs over a very large fpace of country, and where all the arguments, from analogy as well as actual experience, had already been repeatedly canvaffed. And after all, Mrs. Stephens's medicine (whatever might be *her* claim) was really found to be of ufe in relieving the dreadful complaint of the ftone, and is ftill employed, under one form or other, with frequent fuccefs.

The author then reprefents the great enthufiafm which was excited by the introduction of inoculation for the fmall-pox, and the high expectations to which it gave rife—" The divine, the orator, and the phyfician, the eloquent, and the fcientific, alike interefted themfelves, and difplayed their abilities in the caufe of humanity, and the grand jury of Europe pronounced inoculation of the fmall-pox to be a benefit to the human race. But this benefit has proved a phantafma, an illufion merely; the annals of mortality, through half a century, fhew it to be all illufion; and that moft obvious direful impediment to human increafe is ftill unchecked and at large, laughing to fcorn the power of man, and triumphing in its career of peftilential malignity. Now inoculation of the Cow-pox has ufurped the language, and proffers to realize the hope which her elder fifter fo vainly encouraged, *and this beast* is faid to work that miracle which man has failed to accomplifh." We have given here the author's words, as it is right that the favourers of vaccine inoculation fhould know the mode in which their opinions are combated; but, in fober ferioufnefs, we would beg to ask the author, where was the error committed in adopting fmall-pox inoculation with the zeal and enthufiafm with which its firft promoters favoured its claims? The precife objections that are now urged againft the Cow-pox were advanced with ftill more warmth againft variolous inoculation. In particular, it was afferted to be a new and dangerous difeafe, and that it failed in giving a *permanent* fecurity againft the contagion of the difeafe caught in the natural way. And has not it ftood the teft of long experience, and rifen triumphantly above all the attempts to injure its intrinfic worth? Is not the *security* which it affords, moft completely eftablifhed? And has not the celebrated female who introduced it into Europe, a high claim on the gratitude of thofe who, by its means, have at a cheap rate been delivered from the danger of a moft loathfome and deftructive difeafe? It is undifputed, however, that the mortality arifing from the fmall-pox, *in the aggregate*, has increafed fince, and by the introduction of inoculation; and even to fuch a degree as

 fome

ſome years ago to have prevented its adoption into France after mature conſideration. But will it be ſaid that ſuch a wonderful benefit to all individuals who choſe to partake of it, ſhould not have been patronized and adopted on its firſt introduction, becauſe the legiſlature ought to have foreſeen that the prejudice, obſtinacy, and blindneſs of the great maſs of people would, perhaps for centuries, reject the proffered boon? However, be it ſo; at leaſt, the Cow-pox, *this beast* (as the author terms it, with equal elegancy and propriety) is proved without any diſpute to be free from that infectious nature which unfortunately rendered her *elder sister* ſo dangerous to all but her immediate friends, though of human nature.

The more ſober part of our author's treatiſe, however, contains ſome theoretical objections to the probability of the permanent efficacy of vaccination, which, we think, he has himſelf ſufficiently anſwered in the next page by the ſingle obſervation, "that we are too little acquainted with the phyſiological or chemical conformation of the human conſtitution to be able to draw any juſt concluſion from the analogy."——*Facts* follow next, and to theſe we would wiſh to pay every attention. Two, in oppoſition to the efficacy of the Cow-pox, are alledged; the firſt, a declaration of one Robert Newman, whom the author repreſents as a very reſpectable man in a humble ſphere of life, that he had the Cow-pox in Glouceſterſhire when young, and afterwards took the ſmall-pox ſeverely by inoculation. The ſecond is the caſe of a young woman, who early in life had an eruption of a few puſtules on her fingers when employed milking cows, which was then and there called the Cow-pox, who afterwards had ſeveral times reſiſted the ſmall-pox, but at laſt caught it in a very ſevere form. We do not doubt of the fairneſs of the ſtatement on the part of the author, but we hardly think it neceſſary here to point out ſeveral circumſtances which might be ſuggeſted upon theſe repreſentations. The queſtion of the eligibility of the Cow-pox is before the public, the experiments are going on, on a very large ſcale, and our poſterity will judge accordingly.

The author concludes this pamphlet with a propoſal, that in every hundred pariſhes in the kingdom, ten ſubjects ſhould be ſelected for experiment with the Cow-pox; that the magiſtrates, &c. ſhould examine in perſon the operation and the authenticity of the reports; that Dr. Jenner ſhould be retained at an ample ſalary as an Inſpector General, &c. &c.; in ſhort, that the government of the country ſhould take it up as a national concern, and conduct the experiment with the weight of their authority. We have but two objections to make againſt this plan; the one, its utter impracticability, or, at leaſt, the high improbability that it ſhould continue for years to be conducted with the requiſite care and attention; and the other, that the experiment is actually carrying on at an infinitely greater extent, by perſons who have the powerful motive of private character and reputation to prevent them from attempting any ſyſtematical plan of impoſing on the public, to whoſe final determination the whole buſineſs will and *must* be ſubmitted.

An

*An Account of an Ophthalmia which appeared in the Second Regiment of Argyleshire Fencibles in the months of February, March, and April* 1802, *with some Observations on the Egyptian Ophthalmia*; *by* ARTHUR EDMONSTON, Surgeon, 8vo. pp. 33.

THE author in this pamphlet gives an account of an Ophthalmia which occurred in many individuals of the ſecond regiment of Argyleſhire Fencibles, who, in their paſſage to England, were embarked on board the Delft troop ſhip, in a healthy ſtate, which ſhip had been employed in the memorable expedition to Egypt, and on board of which ſome caſes of Ophthalmia had occurred. Several privates of this regiment were attacked on their return to England with Ophthalmia, which diſeaſe they appeared to carry with them in their marches to Colcheſter and Norman Croſs, and which gradually wore itſelf out. The ſymptoms of the Ophthalmia are deſcribed in a clear unaffected manner, and they ſtrongly reſemble thoſe of the true Egyptian diſorder. The mode of cure employed was ſimple and effectual. Scarification of the eye in the beginning, collyria of lead and zinc, bliſters, and inſertion of an opiate ſolution in the eye, in the ſecond ſtage, when the activity of the inflammation had abated. The author aſcribes the diſorder to direct contagion brought from Egypt with the regiment, which opinion he ſupports with effect.

---

*Facts Decisive in favour of the Cow-Pox, including an Account of the Inoculation of the Village of Lowther*; *by* ROBERT JOHN THORNTON, M. D. 8vo. pp. 240.

Dr. Thornton's activity in ſupport of Vaccine Inoculation has been conſpicuous from the beginning of its introduction. The occaſion of the preſent publication was, a ſcheme of Vaccine Inoculation patronized by the late Earl of Lonſdale, in his own place of reſidence and neighbourhood, in the county of Cumberland. After the moſt ample diſcuſſion which this ſubject has received, and the large ſhare of this Journal which we have conſtantly devoted to this intereſting ſubject, it would be uſeleſs to give to our Readers the contents of the preſent work (itſelf a Magazine) formed out of materials collected from every authentic quarter, and in which the direct ſubject profeſſed in the title page, forms but a very ſmall portion of the general contents. Owing, perhaps, to the extenſive influence which the late noble Earl was ſo well known to poſſeſs, the practice of vaccine inoculation ſeems to have met with leſs oppoſition here than in moſt parts of the kingdom, and to have experienced uniform ſucceſs.

---

*A Treatise on the Means of purifying infected Air, of preventing Contagion, and arresting its Progress*; *by* L. B. GUYTON MORVEAU, *Member of the National Institute of France, &c. Translated from the French by R. Hall, M. D.* 8*vo. pp.* 248.

THE ravages committed on the human race by infectious diſeaſes, have always been conſidered as among the direſt calamities to which mankind are expoſed; they depopulate the moſt flouriſhing cities; cities; they dreadfully overbalance many of the natural advantages for commerce and defence, poſſeſſed by ſeveral of the moſt important ſettlements; they ſpread alarm, terror, and confuſion, wherever they appear, and oppoſe the exerciſe of one of the moſt important of the ſocial duties, by rendering the attendance on the ſick either an heroic ſacrifice to affection, which few can make, or a mercenary ſervice which muſt be made amply to repay the perſonal riſk.

Few ſubjects, therefore, have a greater claim to public and national attention, than the diſcovery of any mode of leſſening or preventing theſe formidable evils; and the ſenſe of common danger has induced the governing powers of almoſt every civilized country, from the earlieſt ages, to eſtabliſh regulations (more or leſs ſevere according to the exigency of the caſe) in order to prevent the ſpreading of infectious diſeaſe.

We have no heſitation in aſſerting, that the method of fumigation with the vapour of the more powerful acids affords a moſt important corrective of infection, the value of which has already been aſcertained by numerous and extenſive trials, and is therefore deſerving of the higheſt attention.

The object of the author in the work before us, is to examine into this ſubject at large, to aſſert his claim to a priority of diſcovery, by inconteſtable proofs, and to eſtabliſh the ſuperiority of the muriatic acid over other ſubſtances of a ſimilar nature, which have been employed in different trials. The hiſtory of the diſcovery and employment of acid vapours in checking infection is curious. The uſe of fumigations of almoſt every kind of combuſtible ſubſtance, is a practice of great antiquity, and has formed a regular part of the practice in various Hoſpitals and Lazarettoes for the plague for many centuries: even the vapour of vinegar appears to have been long employed, and the tranſition from theſe ſubſtances to the uſe of the mineral acid vapours, appears ſo obvious, that we may be ſurprized that it had remained ſo long undiſcovered. The late Dr. Johnſtone of Worceſter, (a man of a very acute mind, and a warm zeal for the improvement of his profeſſion) was certainly the firſt who is known to have employed the vapour of the muriatic acid in correcting (very ſuccesſfully) the contagion of a very malignant fever which had appeared at Kidderminſter in 1756, and had ſhewed ſuch peculiar virulence, as to have acquired the name of "the Kidderminſter fever." * This ſolitary fact, though ſo important, was ſoon neglected, and nothing more appeared on the ſubject, till in the year 1773, M. Morveau-Guyton employed the ſame acid to fumigate the cathedral church of Dijon, which had been made inſupportably infected by the putrefaction of a number of corpſes which it was neceſſary to remove; and alſo, in the ſame year, a few months afterwards, the ſame eminent chemiſt repeated the fumigation in the jails of Dijon, which were then infected

* Dr. Johnſtone publiſhed an account of this fever, and the mode of fumigation, in a Treatiſe, entitled An Hiſtorical Diſſertation on the Malignant Fever which prevailed at Kidderminſter, in 1756.

infected with a very malignant fever. It will be proper to obferve here, that we can fcarcely entertain any doubt that this celebrated chemift and excellent philofopher propofed this method of fumigation from his own reafoning on the fubject; for the Journals and other publications of that time fhew, that the properties of the *muriatic acid gas* were then engaging the attention of the chemical world; and the ftriking experiment of its union with, and thereby neutralizing *ammoniacal gas*, had often been performed. Now, as ammonia was then known to be difengaged by putrefaction, it was certainly a highly ingenious idea of this chemift to let loofe in putrefying air a quantity of muriatic acid gas, in order to correct the ammonia, in the production of which the putrid procefs was thought principally to confift.

In 1774, the muriatic fumigation (which had hitherto made but little progrefs) was employed, at the recommendation of the late *Vicq d'Azyr* to purify ftables from the infection of a very deftructive malady, which, at that time, proved fatal to a vaft number of cattle in the fouth of France. In 1780, the Academy of Sciences, (who had been confulted by government on the beft means of correcting the infalubrity of prifons), appointed a committee for this purpofe, compofed of Meffrs. *Duhamel, De Montigny, Leroi, Tenon, Tillet*, and *Lavoisier*, all men of high reputation and ability, who, among other means, recommend in ftrong terms, the method of muriatic fumigation fo fuccefsfully employed by M. Morveau.

Still, however, the progrefs made in the general introduction of this fumigation was very flow and imperfect; but the dreadful epidemic at Genoa in 1799, and the ftill more fatal and extenfive malady which foon after ravaged Andalufia, and efpecially the town of Cadiz, (which our readers will recollect, from the circumftance of its being then blockaded by the Britifh Fleet) obliged the governments to have recourfe to every vigorous means of correcting the infection, and, among thefe, the muriatic fumigation was employed with eminent advantage.

The author of this treatife proceeds to notice the very important experiments made in this country, by defire of the Lords of the Admiralty, with the *nitrous* acid vapour, on board the Union Hofpital fhip in Nov. 1795, to correct the contagion of a very malignant fever, which had made great ravages on board the Ruffian fhips at Sheernefs. The trial of this acid vapour was made, (as our Readers muft well know), at the fuggeftion of Dr. Carmichael Smyth; and the fuccefs was fo complete, as to leave no reafon to doubt of the high efficacy of this fumigation. Subfequent trials have confirmed this opinion, and have induced the Houfe of Commons to vote a reward to Dr. Smith, for this valuable and eafy method of deftroying the contagion of infectious fevers.

In the third fection of this very interefting work, M. Guyton introduces a number of comparative experiments, made with a view of determining what fubftances are the moft effectual in correcting the fetor of air in which meat has long putrified. For the particulars of thefe valuable experiments we muft refer the reader to the work itfelf, as well as for feveral chemical arguments which cannot be eafily eaſily given in a condenſed form. Suffice it to ſay, that the various odorous reſins, &c. burnt in the infected air, produced no other effect than imperfectly to conceal the fetor of the air itſelf, and this fully eſtabliſhes the opinion (which indeed is now generally received) that merely to ſcent an infectious air with any ſtrong perfume, takes away *none of its noxious properties*, and in general, does much harm in hoſpitals, and other large ſources of infection, by rendering the offenſive ſmell for a while tolerable, and therefore often ſupplying the place of thorough ventilation, or the more powerful fumigations, which appear actually to *deſtroy* the fetid particles by chemical combination. Of this latter kind of fumigation are all the acid vapours. The acid gaſes employed in the experiments were, the ſulphureous, the nitric, the muriatic, and the oxygenated muriatic, and the power of the three latter by far exceeded that of the former. The author then attempts to inſtitute a compariſon between theſe three acids, with regard to the power which they poſſeſs in correcting infectious air. We cannot ſay that he has eſtabliſhed, in a very ſatisfactory manner, the point which he endeavours to prove, namely, the ſuperior efficacy of the muriatic and the oxygenated muriatic acids over the nitrous. The difficulties which lie in the way of ſuch a compariſon are very great and obvious, and probably it would require the experience of many years to eſtabliſh the point. The author lays ſome ſtreſs on the circumſtance, that Dr. Smyth thought it proper to fumigate much oftener with the nitrous vapour, than the French and Spaniſh phyſicians did with the muriatic. But it is evident, that in neither caſe could it be at all inferred, *how much* of the vapour was requiſite; and where the method of employment is ſo cheap and eaſy of application, no great harm can ariſe from exceſs of precaution, at all comparable to the conſequences of neglect. A more valid argument in favour of the muriatic acid is its much greater *diffuſibility* than the nitrous, and its requiring a much leſs heat to be expelled in a gaſeous form from the mixture of oil of vitriol and ſalt, than the nitrous, from oil of vitriol and nitre. However, it is by no means neceſſary to determine a preference between the two, ſince the anti-putreſcent powers of both are ſo very powerful and extenſive, that *either* of them may be reſorted to with the higheſt confidence of ſucceſs, and we earneſtly wiſh to ſee this method of fumigation enjoined in every jail, hoſpital, and fever houſe in the kingdom, where the virulence of infection reſiſts the common methods of ventilation and cleanlineſs.

A conſiderable part of this work is taken up with an attempt to extend the ſuppoſed operation of oxygen upon the animal ſyſtem to a great variety of circumſtances; and in the true ſpirit of modern chemiſtry, to explain the operation of medicines, and the effect of morbid cauſes, by the laws of chemical affinity, which are aſcertained in the laboratory. We ſhall not treſpaſs on our Readers time by detailing every crude notion and vague conjecture to which this method of philoſophizing on the laws of the *living* animal has given riſe. In the inſtance before us, we owe ſo much to the *ſtrength* of chemiſtry that we ſhall forbear to expoſe its *weakneſs*.

